# Automatic delineation of malignancy in histopathological head and neck slides

**DOI:** 10.1186/1471-2105-8-S7-S17

**Published:** 2007-11-01

**Authors:** Mutlu Mete, Xiaowei Xu, Chun-Yang Fan, Gal Shafirstein

**Affiliations:** 1 Department of Applied Science, University of Arkansas at Little Rock, Little Rock, Arkansas, USA; 2 Department of Information Science, University of Arkansas at Little Rock, Little Rock, Arkansas, USA; 3 Department of Pathology, University of Arkansas for Medical Sciences, Little Rock, Arkansas, USA; 4 Department of Otolaryngology, University of Arkansas for Medical Sciences, Little Rock, Arkansas, USA

## Abstract

**Background:**

Histopathology, which is one of the most important routines of all laboratory procedures used in pathology, is decisive for the diagnosis of cancer. Experienced histopathologists review the histological slides acquired from biopsy specimen in order to outline malignant areas. Recently, improvements in imaging technologies in terms of histological image analysis led to the discovery of virtual histological slides. In this technique, a computerized microscope scans a glass slide and generates virtual slides at a resolution of 0.25 μm/pixel. As the recognition of intrinsic cancer areas is time consuming and error prone, in this study we develop a novel method to tackle automatic squamous cell carcinoma of the head and neck detection problem in high-resolution, wholly-scanned histopathological slides.

**Results:**

A density-based clustering algorithm improved for this study plays a key role in the determination of the corrupted cell nuclei. Using the Support Vector Machines (SVMs) Classifier, experimental results on seven head and neck slides show that the proposed algorithm performs well, obtaining an average of 96% classification accuracy.

**Conclusion:**

Recent advances in imaging technology enable us to investigate cancer tissue at cellular level. In this study we focus on wholly-scanned histopathological slides of head and neck tissues. In the context of computer-aided diagnosis, delineation of malignant regions is achieved using a powerful classification algorithm, which heavily depends on the features extracted by aid of a newly proposed cell nuclei clustering technique. The preliminary experimental results demonstrate a high accuracy of the proposed method.

## Background

Histopathology remains one of the most critical steps in the diagnosis and treatment of virtually any kind of cancer. The incidence of cancer has remained fairly constant since the early 1990's, with an average of 486.6 new cases per 100,000 Americans documented in 2002 [1]. Mortality from all types of cancer in 2002 was 288,763 [1], and remains the second leading cause of death in the United States [2]. In the U.S., nearly 37,000 men and women were diagnosed with head and neck cancer in 2003 [1]. Head and neck cancers are highly fatal, and survival rates have not decreased notably over time [1].

The treatment for many types of cancer, including head and neck cancers, consists of surgical removal followed by histopathologic examination. In the diagnosis of head and neck cancer, tumor biopsy is one of last phases after a group of preliminary check such as historical, physical and non-invasive examination. Basically, biopsy procedure involves excising region-of-interest and sending the specimen to the histopathologist for detailed investigation under microscope. Although biopsies, especially the ones that are deep inside the body or grew in problematic-to-operate locations, are painful for the patients, they are important for cancer classification.

One major factor that affects the prognosis of patients with head and neck cancer is regional lymph node metastases, the spread of malignant cells from primary site. Accordingly, sentinel lymph node biopsy (SLNB) is broadly accepted as a first, more convenient and less painful way of prognosis. Thus, histopathologists investigate not only biopsy of head and neck tumor but also biopsy of lymph nodes. Accurate evaluation is required for neck management and improvement of head and neck cancer patient survivals.

Traditionally, selective neck lymph node dissection is essential for neck metastasis evaluation and this procedure requires removal of many lymph nodes in several regions, which is associated with increased morbidity, such as spinal accessory nerve dysfunction and related shoulder disability. In an attempt to avoid unnecessary treatment to the clinically negative neck and thus, decrease morbidity, the sentinel lymph node biopsy technique (SLNB), a minimally invasive technique, is increasingly used in the place of selective neck lymph node dissection and is emerging as a successful means of identifying occult malignant cells of neck lymph nodes in patients with head and neck cancer. The Second International Conference on Sentinel Node Biopsy in Mucosal Head and Neck Cancer held in September 2003 (Zurich, Switzerland) has sufficiently validated SLNB as a useful approach of neck staging for early head and neck cancer based on multi-center studies. At the conference, the use of conventional haematoxylin and eosin (HE) staining on step-sections of the entire node cut at intervals of 15 μm is recommended consequently.

For this particular reason, histopathologic examination is mandatory in selective and suspicious cases. But, tedious work required for the preparation and review of many histological sections (ranging from dozens to hundreds depending on the size of tissue) is a notable limiting factor for generalized adoption of this potentially very useful technique in pathology laboratories. Additionally, some rural areas may not have enough pathologists for examining large number of slides. As a result, automated systems such as computer aided diagnosis (CAD) that enable accurate screening of large number of digital slides, and report the suspicious slides for further examination by the pathologists would be a significant help for histopathologists.

Recent improvements in imaging techniques led to discovery of virtual histological slides, where an automated light microscope [3] scans a glass slide and generates a virtual slide at a resolution of 0.25 μm/pixel. Output images are of sufficiently high quality to attract immense interest within the research community. High-resolution medical imaging technique combined with pattern recognition has led to a new research area: tumor delineation in high-resolution histopathologic slides. To the best of our knowledge, there is no existing study in the literature addressing high-resolution, wholly-scanned histopathologic slide understanding in the context of CAD. When compared with other types of medical images, computer-assisted tumor identification in histopathological slides is emerging branch in both medical and computer sciences.

In conjunction with traditional histological review by pathologists, recognition of cancer cells in histopathologic slides generated en masse and cheaply by special high-resolution scanners has great potential to improve the efficiency and accuracy of evaluating cancer in primer location and sentinel lymph node. High-speed slide scanners automatically generate much larger number of tissue sections from any single tissue at smaller intervals, thus can increase the sensitivity of overall system by detecting small malignant cells that would otherwise escape from human eye. As expected, the larger cut interval, the lower chance histopathologists can investigate whole malignancy using current techniques.

**Figure 1:**
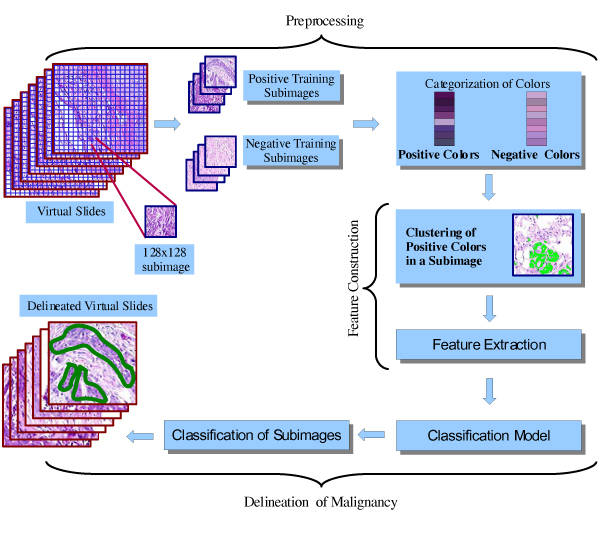
**General schema of proposed framework**. The proposed framework consists of data preprocessing (Processing), feature construction and delineation of malignancy steps. In the processing step, the virtual slides are partitioned into equi-size subimages of 128 × 128. The subimages are labeled to positive (malignant) or negative (healthy) by human pathologist. Then a subset of subimages is selected as training data. Furthermore, a color analysis to categorize colors is performed. In the feature construction step, density-based clustering algorithm is applied to find positive color areas, which builds the features for the classification of subimages. In the step of delineation of malignancy, a classification model is trained. The trained model is then used to classify subimages. The area of malignancy is then delineated.

In this study we conduct a pioneering work to determine how accurately a computer can be trained to recognize small tumor cells in high-resolution images. Introduced framework can be seen in Figure 1. Thanks to high-resolution digital microscopes, the framework scans and evaluates hundreds of histological sections of tumor at much higher efficiency. In this study a quite common staining procedure, namely haematoxylin and eosin (HE), is chosen to dye slides. As invasive head and neck tumor cells differ from normal tissue regarding cancer cell morphologies, we particularly concentrate on distinguishing cell irregularities and color differences in cells. Image understanding using subimages is more practical for machine learning approaches since malignant regions show similar characteristics in small scale. Hence, we introduce a subimage-based annotation system using local features rather than global features. Therefore, the first step in the data preparation is to partition every slide into subimages. In general, the framework learns from subimages that are labeled as positive and negative by pathologist; and attempts to identify areas that contain tumor cells. The main advantage of this model is to guide pathologists through their decisions on histopathological slides by exploiting high throughput of whole slide imaging systems. When compared with other histopathological image understanding scenarios [4-7], and [8] which only look for cell anomalies locally, whole-slide processing exploiting cluster-based features makes our avenue unique in this domain. In addition, thanks to this innovative approach and parallel DBSCAN [9], hundreds of slides can be examined at one time in a grid environment [10].

## Results and discussion

In this section, we first describe the datasets used for the evaluation of our framework. We then report the performance using standard measures. Additionally we analyze the results and give a discussion of the performance of the system.

**Figure 2:**
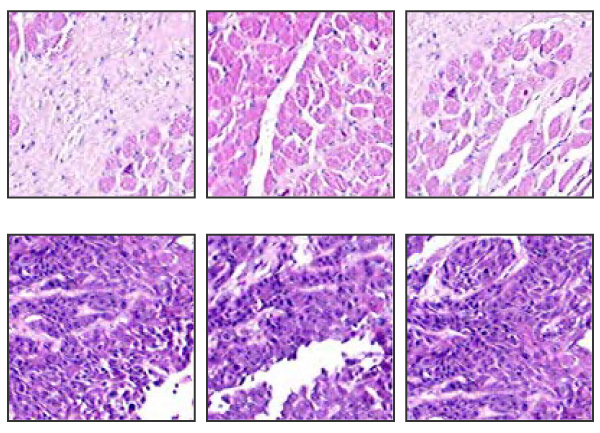
**Three samples from negative (first row) and positive (second row) training subimages**. The negative image typically has light red color and low density of cells, while the positive image is characterized by dark color and high density of cells.

In our study we use seven histopathological slides. Four slides have malignant areas that are delineated by pathologist. On the other hand, no malignant area is assessed in three slides. Subsequently, four whole slides with malignant regions and the other three healthy slides are labeled as positive (P) and negative (N) respectively. Table 1 summaries image resolutions for slides used in this study. Each whole slide is further divided into subimages. The size of these subimages is fixed to 128 × 128. Examples of such subimages are shown in Figure 2. The first row shows three negative subimages; and the second row shows three positive subimages. We collect 327 positive (malignant) and 373 negative (healthy) subimages from slide P_1 _and 255 negative subimages from slide N_2_. To delineate cancer areas, we train a two-class support vector machine (SVM). The trained SVM is then tested on the remaining subimages in all seven histopathological slides.

**Table 1:** Size of seven virtual histopathological slides

**Slide ID**	**Width**	**Height**
P_1_	7396	8408
P_2_	8811	8718
P_3_	7506	11672
P_4_	7419	4994
N_1_	4906	7253
N_2_	6895	6485
N_3_	9058	7845

We evaluated the performance of proposed framework by recall, precision and predictive accuracy that are based on true positive (TP), false positive (FP), true negative (TN), false negative (FN) in terms of subimages. To avoid confusion with the terms used to evaluate our system, we refer the reader to confusion matrix. For a binary classification problem like this study, the elements of the matrix are shown in Table 2. Precision (specificity), recall and accuracy are very common measures in binary classifications. Precision is defined as the percentage of positive predictions that are correct, and calculated by TPTP+FP
 MathType@MTEF@5@5@+=feaafiart1ev1aaatCvAUfKttLearuWrP9MDH5MBPbIqV92AaeXatLxBI9gBaebbnrfifHhDYfgasaacH8akY=wiFfYdH8Gipec8Eeeu0xXdbba9frFj0=OqFfea0dXdd9vqai=hGuQ8kuc9pgc9s8qqaq=dirpe0xb9q8qiLsFr0=vr0=vr0dc8meaabaqaciaacaGaaeqabaqabeGadaaakeaadaWcaaqaaiabdsfaujabdcfaqbqaaiabdsfaujabdcfaqjabgUcaRiabdAeagjabdcfaqbaaaaa@3490@. On the other hand, recall (sensitivity) is known as the percentage of positive labeled instances that were predicted as positive and found by TPTP+FN
 MathType@MTEF@5@5@+=feaafiart1ev1aaatCvAUfKttLearuWrP9MDH5MBPbIqV92AaeXatLxBI9gBaebbnrfifHhDYfgasaacH8akY=wiFfYdH8Gipec8Eeeu0xXdbba9frFj0=OqFfea0dXdd9vqai=hGuQ8kuc9pgc9s8qqaq=dirpe0xb9q8qiLsFr0=vr0=vr0dc8meaabaqaciaacaGaaeqabaqabeGadaaakeaadaWcaaqaaiabdsfaujabdcfaqbqaaiabdsfaujabdcfaqjabgUcaRiabdAeagjabd6eaobaaaaa@348C@. Lastly, the predictive accuracy of the classifier is calculated as TP+TNTP+TN+FP+FN
 MathType@MTEF@5@5@+=feaafiart1ev1aaatCvAUfKttLearuWrP9MDH5MBPbIqV92AaeXatLxBI9gBaebbnrfifHhDYfgasaacH8akY=wiFfYdH8Gipec8Eeeu0xXdbba9frFj0=OqFfea0dXdd9vqai=hGuQ8kuc9pgc9s8qqaq=dirpe0xb9q8qiLsFr0=vr0=vr0dc8meaabaqaciaacaGaaeqabaqabeGadaaakeaadaWcaaqaaiabdsfaujabdcfaqjabgUcaRiabdsfaujabd6eaobqaaiabdsfaujabdcfaqjabgUcaRiabdsfaujabd6eaojabgUcaRiabdAeagjabdcfaqjabgUcaRiabdAeagjabd6eaobaaaaa@3E1C@.

**Table 2:** Confusion matrix

		Classified	
		
		Malignant (+)	Healthy (-)
Actual	Malignant (+)	TP	FN
	Healthy (-)	FP	TN

Table 3 shows predictive accuracy obtained on four positive slides. The trained SVMs achieved excellent result in terms of accuracy. It accurately identified subimages with malignancy in an accuracy of at least 94%. The average accuracy of detecting malignant subimages is 96.25%, which is much higher than 72.3% achieved by Zhao et al. [11] for classification histological images. The achieved accuracy is also significantly better than that of Diamond et al. [12], who proposed a machine vision system for the classification histological slides, which achieved an accuracy of 79.3% in the classification of tumor regions by using shape and Haralick [13] features. Furthermore, as one can see from the results in Table 3, the trained SVM model also obtained good results with respect to the recall and precision. The average recall on four positive slides is 85.75%; and the precision is 80.75%. That means the system can find most malignant areas (or subimages) accurately.

**Table 3:** Accuracy for four malignant slides

Slide	Recall	Precision	Accuracy
P_1_	88%	92%	97%
P_2_	78%	75%	94%
P_3_	88%	85%	95%
P_4_	89%	71%	99%

**Figure 3:**
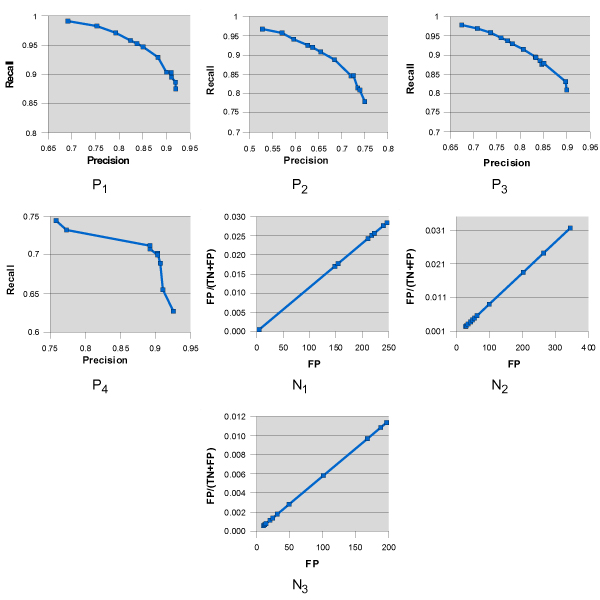
**Results on seven slides**. The precession and the recall are plotted for positive slides (P_1_, P_2_, P_3 _and P_4_). The false positive rate FP/(TN+FP) and false positive (FP) are shown for negative slides (N_1_, N_2_, and N_3_)

Since FN and FP ratio are the major concern for CAD systems, we conducted a set of experiments to investigate the performance in terms of precision and recall. The plots of recall vs. precision for positive slides were depicted in Figure 3 (P_1_–P_4_). As one can see, the trained SVM achieve good precision and recall on all positive slides. Again it demonstrates that the proposed method can accurately detect most malignant areas. For the negative slides, it is important to minimize the false positive. We use FPTN+FP
 MathType@MTEF@5@5@+=feaafiart1ev1aaatCvAUfKttLearuWrP9MDH5MBPbIqV92AaeXatLxBI9gBaebbnrfifHhDYfgasaacH8akY=wiFfYdH8Gipec8Eeeu0xXdbba9frFj0=OqFfea0dXdd9vqai=hGuQ8kuc9pgc9s8qqaq=dirpe0xb9q8qiLsFr0=vr0=vr0dc8meaabaqaciaacaGaaeqabaqabeGadaaakeaadaWcaaqaaiabdAeagjabdcfaqbqaaiabdsfaujabd6eaojabgUcaRiabdAeagjabdcfaqbaaaaa@3470@ as measure of false positive rate, which should be minimized. We computed FPTN+FP
 MathType@MTEF@5@5@+=feaafiart1ev1aaatCvAUfKttLearuWrP9MDH5MBPbIqV92AaeXatLxBI9gBaebbnrfifHhDYfgasaacH8akY=wiFfYdH8Gipec8Eeeu0xXdbba9frFj0=OqFfea0dXdd9vqai=hGuQ8kuc9pgc9s8qqaq=dirpe0xb9q8qiLsFr0=vr0=vr0dc8meaabaqaciaacaGaaeqabaqabeGadaaakeaadaWcaaqaaiabdAeagjabdcfaqbqaaiabdsfaujabd6eaojabgUcaRiabdAeagjabdcfaqbaaaaa@3470@ versus FP for negative slides since TP equals zero for those slides. In this setting, note that (TN+FP) gives exactly the number of subimages in the slides and is constant during experiments, so that FPTN+FP
 MathType@MTEF@5@5@+=feaafiart1ev1aaatCvAUfKttLearuWrP9MDH5MBPbIqV92AaeXatLxBI9gBaebbnrfifHhDYfgasaacH8akY=wiFfYdH8Gipec8Eeeu0xXdbba9frFj0=OqFfea0dXdd9vqai=hGuQ8kuc9pgc9s8qqaq=dirpe0xb9q8qiLsFr0=vr0=vr0dc8meaabaqaciaacaGaaeqabaqabeGadaaakeaadaWcaaqaaiabdAeagjabdcfaqbqaaiabdsfaujabd6eaojabgUcaRiabdAeagjabdcfaqbaaaaa@3470@ changes linearly, as can be seen in of Figure 3 (N_1_–N_3_). The false positive rate is very low, which is less than 10% in average. It is known that a high precision normally means a low recall or sensitivity. Our method achieves good recall without compromise too much on the precision.

**Figure 4:**
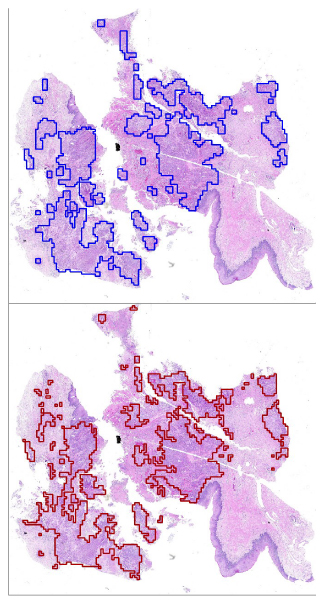
**Visual comparison of the result of proposed framework (above) and the assessments of the histopathologist (below) for slide P_2_**. The proposed method picks all major malignancies with very few false positives.

Additionally, a visual comparison of the pathologist's assessment and predicted tumor region by our framework for slide P_2 _was shown in Figure 4. It shows that there is a good match between the pathologist's assessment and the outcome of our method.

**Figure 5:**
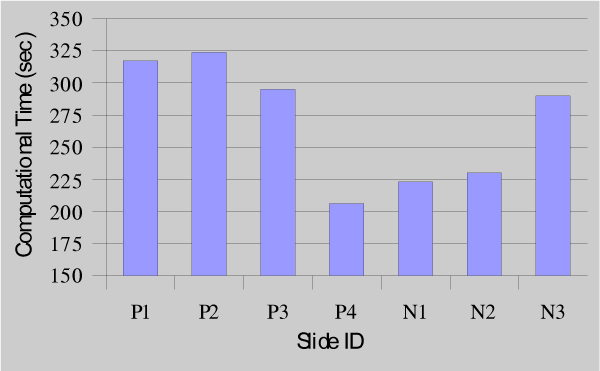
**Average runtime for seven virtual slides**. The average total process time for all 7 slides is depicted using the bars. The time is measured by running time in second.

We measured the running time for our method on Pentium 4 2.4 GHz workstation equipped with 1 GB memory. Average runtime to completely draw region-of-tumor, which includes all the phases of the method is presented in Figure 5. As one can see that the negative slides comparably take less time than that of positive ones due to the fact that there are usually more positive areas in positive slides; and it took more time to outline positive areas than negative areas.

## Conclusion

In this paper, we presented a new algorithm to detect tumors by using one of the latest imaging technologies, virtual histological slides. We studied a total of seven wholly-scanned neck and cancer tissues. With the intention of having best classification accuracy on the problem of squamous cell carcinoma detection, we exploited density-based clustering algorithm DBSCAN [14] for feature extraction in equi-size subimages, which are obtained from the systematic partitioning of original slides. The delineation of malignant regions is achieved by using a powerful classification algorithm, namely SVMs, that depends on the features extracted by using our newly proposed cell nuclei clustering technique, which plays an important role in the discussed framework. We chose recall, precision, and predictive accuracy as the evaluation criteria. The experiment results validate our framework by providing an average accuracy of 96% on all positive slides and low ratio of false positives on all negative slides. To the best of our knowledge, this is the first work that deals with wholly-scanned histopathological slides at the cellular level targeting CAD applications.

Future work will be more on the dependency analysis between cancer and healthy areas. It seems that the current framework can be applied not only to the classification of head and neck cancer, but also to other type of cancers, such as breast and colon cancer. We will investigate the applicability of our framework for various types of cancer in the future.

## Methods

### Experimental data

In this section, we briefly describe histopathological slides we used in this research. We work on seven slides of different specimens that are stained with haematoxylin and eosin (HE), which is the standard staining in routine histopathology, and captured with 20× objective lens. Four slides have malignant areas that are delineated by pathologist. On the other hand, no malignant area is assessed in three slides. Subsequently, four whole slides with malignant regions and the other three healthy slides are labeled as positive and negative respectively. Table 1 summaries image resolutions for slides used in this study.

### Data preparation

The major goal of the examination of the histological slides is to detect tumor region if exists any. The boundary of malignant region is also crucial for the margin analysis of the slide. Hence, the main task of this study is to delineate malignant areas in addition to determining if the slide contains any cancer region. With the help of the practice of the histopathology, the size of the subimages is fixed to 128 × 128. A subimage with this size was found to be both large enough to include malignancy of clinical interest as well as small enough to demonstrate abnormal cell growing. Training dataset is captured at random points in two slides. We collect 327 positive (malignant) and 373 negative (healthy) subimages from slide P_1 _and 255 negative subimages from slide N_2_. Please note that during arbitrary selection of training subimages it is crucial to choose subimages that present a wide range of positive and negative structures. Thus, care must be taken in the stage of training dataset construction; otherwise, misleading subimages would affect negatively the classifier model. Because annotated areas by pathologist generally differ in density of nuclei, normal tissue, lipid, and air, we avoid selecting fuzzy subimages and try to capture clear-to-classify subimages. In Figure 2, we illustrate six training images, three negatives and three positives on the first and second line respectively.

The histological images in this study are stained with HE. We remove some of the artifacts from each slide due to the fact that manual preparation of images some artifacts are quite common. Especially the dark blue-purple spots of haematoxylin are detected in four slides and considered as color artifacts by the pathologist.

**Figure 6:**
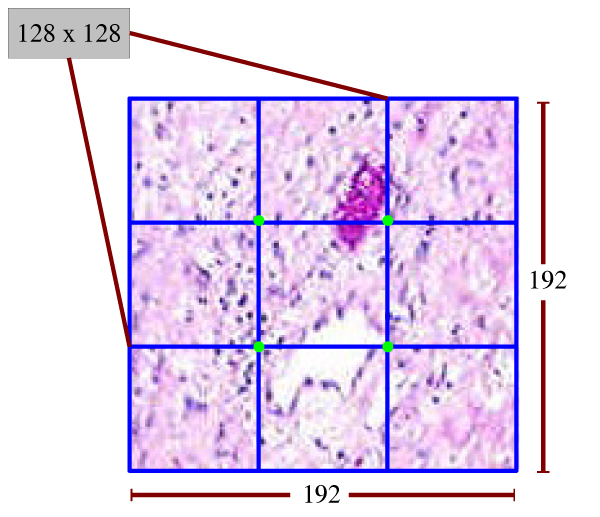
**Subimage layout for a 192 × 192 image that is partitioned into four blocks**. This figure shows an example of how a 192 × 192 image is partitioned into four 128 × 128 subimages. The four green points are the centers of the 4 subimages. Each subimage has a 50% overlap with another subimage.

In order to delineate tumor regions, each slide is partitioned in such a way that each N × N subimage has a half overlap with the next N × N subimage in four directions. Incomplete subimages on the last column or row are ignored. The size of each subimage is fixed at same size with training subimages, which is 128 × 128. Figure 6 depicts an example layout for a 192 × 192 image, which is partitioned into four blocks. Each of four green dots in the image represents the center of its squared corresponding subimage. Unlike some CAD systems, in the data preparation stage, we apply neither histogram equalization nor filtering.

### Feature design

The main task of this study is to develop an algorithm (or classifier) to classify the subimages to two classes: positive (or malignant) or normal (or healthy). The effectiveness of the current classifiers is inherently limited by the features extracted to represent the subimages in the virtual slides. Even though color based features provides valuable information for visual perception, it will not be sufficient to rely on only colors as features for subimage classification. This is because manual assessment of histological slide depends not only on color properties of stained material but also on structures of uncontrolled growth of tumor cells.

There is a substantial body of literature on feature selection algorithms used in image analysis. One of these studies [15] comprehensively evaluated feature selection, extraction, and construction. The authors shed light on approaches and algorithms from a data mining perspective. Moreover, Evgeniou et al. [16] investigated the role of image representation using kernel machine classifier specifically SVMs [17]. As choosing too many features can decrease the efficiency of machine learning, in the same way, insufficient number of features can be primary reason for low accuracy rate.

Haematoxylin and eosin (HE) staining is a well-known method in histology. In the case of histological slides, the process of staining digs corrupted nuclei out and makes the inspection of tumor regions easier. HE staining is an application of the basic dye haematoxylin, which colors nucleic acids (ribosome and cell nucleus etc.) with blue-purple hue, and eosin, which makes cytoplasm and proteins bright pink. These functionalities highlight essence of color selection and lead us to concentrate on color as well as irregular and connected shape of nuclei. As for variety in HE staining techniques in different laboratories, please note that the study presented in the paper would be more robust with the use of automated slide staining machines that provide evenly stained slides. Texture based features are also taken into consideration in the phase of feature design since they are proven to often increase accuracy of computer vision systems. In contrast, we reached that high resolution subimages do not show homogeneity in terms of malignancy. Hence it is concluded that texture features would not be discriminative in the recognition of tumor areas due to the high-resolution property of experimental slides.

### Characterization of malignant colors

Unlike some earlier studies [4,7,8], we opt to exploit every single pixel. The native color space of virtual histological slides is RGB (Red, Green, and Blue). However, it is not often desired for color image understating since it does not correspond well to the visual perception. The more proper color space for human color sensitivity is HSV (Hue, Saturation, and Value). The reason is that spatial differences are more similar to the variations between colors seen by human [18]. Moreover in contrast to RGB, HSV is highly resistant to illumination changes. HSV components, hue, saturation, and value, can be interpreted as outcome, percentage of white, and brightness respectively. Consequently, RGB values of a pixel are mapped to HSV space, which represents primitive values for our classification approach. Based on training subimages, a color analysis to build a global pixel classifier is carried out by considering frequency of each color *c*.

Definition 1: Let *c *= [*h*, *s*, *v*] denote a unique color from HSV color space *H*; *C*_*P *_and *C*_*N *_be set of all unique colors obtained from positive and negative subimages, respectively, i.e.:

*C*_*P *_= {*c*| *c *appears in positive subimages}, and *C*_*N *_= {*c*| *c *appears in negative subimages}.

Definition 2: Let *X *and *Y *be discrete random variables representing non-zero probability of colors for the set of *C*_*P *_in positive training subimages and the set of *C*_*N *_in negative training subimages, respectively.

Definition 3: The set of p*ositive distinctive colors *(PDC) which indicates likely malignant regions is formulated as *PDC *= {*c *| (*c *∈ *C*_*P*_) ∧ (*f *(*c*) ≥ *t*) ∧ (*D*_*KL *_(*P *(*X *= *c*)|| *P *(*Y *= *c*)) > *T*)} where, *f*(*c*) indicates frequency of color c in all training subimages and is conditioned to be greater than *t*; and *D*_*KL *_(*P *(*X *= *c*) || *P*(*Y *= *c*)) is Kullback-Leibler [19] divergence of probability of color *c *in negative training subimages from probability of color *c *in positive subimages. More formally, the divergence is defined to be

DKL(P(X=c)||P(Y=c))=P(X=c)log⁡2P(X=c)P(Y=c).
 MathType@MTEF@5@5@+=feaafiart1ev1aaatCvAUfKttLearuWrP9MDH5MBPbIqV92AaeXatLxBI9gBaebbnrfifHhDYfgasaacH8akY=wiFfYdH8Gipec8Eeeu0xXdbba9frFj0=OqFfea0dXdd9vqai=hGuQ8kuc9pgc9s8qqaq=dirpe0xb9q8qiLsFr0=vr0=vr0dc8meaabaqaciaacaGaaeqabaqabeGadaaakeaacqWGebardaWgaaWcbaGaem4saSKaemitaWeabeaakiabcIcaOiabdcfaqjabcIcaOiabdIfayjabg2da9iabdogaJjabcMcaPiabcYha8jabcYha8jabdcfaqjabcIcaOiabdMfazjabg2da9iabdogaJjabcMcaPiabcMcaPiabg2da9iabdcfaqjabcIcaOiabdIfayjabg2da9iabdogaJjabcMcaPiGbcYgaSjabc+gaVjabcEgaNnaaBaaaleaacqaIYaGmaeqaaOWaaSaaaeaacqWGqbaucqGGOaakcqWGybawcqGH9aqpcqWGJbWycqGGPaqkaeaacqWGqbaucqGGOaakcqWGzbqwcqGH9aqpcqWGJbWycqGGPaqkaaGaeiOla4caaa@5C36@

The Kullback-Leibler divergence, introduced by S. Kullback and R.A. Leibler in 1951 [19], can be considered as a kind of a distance that quantifies the expected amount of color *c *information for discrimination between the two probability densities. Note that the Kullback-Leibler divergence is not symmetric; hence, is not a metric. In same manner we define the set of *negative distinctive colors *(NDC) that generally indicate non-malignant regions as

*NDC *= {*c *| (*c *∈ *C*_*N*_) ∧ (*f*(*c*) ≥ *t*) ∧ (*D*_*KL *_(*P *(*Y *= *c*) || *P*(*X *= *c*)) > *T*)}.

### Color generalization

In the previous section we defined positive distinctive colors PDC, by which possible malignancy is represented. Recall that the set of PDC is obtained by considering randomly sampled images labeled by the pathologist. Although we start with large number of subimages, arbitrary selection can raise the question about underrepresented colors. Accordingly it cannot be guaranteed that a particular color *c *that is densely surrendered by elements of PDC would belong to PDC. In other words, we might coincidentally miss some positive indicative colors while discovering PDC.

Example 1 The color [275, 100, 51], namely indigo, by chance, was never encountered in positive training subimages and therefore does not belong to PDC, despite the fact that the neighbors of color indigo, for instance, [276, 100, 51], [275, 99, 51], [275, 100, 49], and [275, 100, 52] were all elements of PDC, which means that indigo could be actually a good indicator of positive distinctive color.

In order to avoid this and similar problems that may occur in the phase of subimage classification, we draw color boundaries in HSV space using decision tree induction [20]. By doing this we ensure that the underrepresented colors like indigo would be elements of PDC.

Definition 4: Let *M *: *c *→ *l *∈ {*positive*, *negative*} be a decision tree classifier induced from PDC and NDC; and maps a color of pixel to a class label, positive (malignant) or negative (healthy).

The reason to prefer the decision tree was its interpretability, where generated rules can be interpreted easily. Besides, the tree allows one to detect the set of rules and particularly the most important features in the decision-making. All colors of PDC and NDC are used as positive and negative labeled instances through decision tree induction. Widely used implementation of decision tree, c4.5 [20], was employed for color classification (Definition 4). Ten-fold cross-validation on training dataset (PDC and NDC) results in correct classification rate of 99.4%.

It is shown that the rules extracted from obtained tree comply with the color interpretation of histopathology. The component of hue is more informative than the saturation or value to distinguish malignant and healthy regions. It is important to bear in mind that positive colors mentioned in the rest of the paper refer those colors that are classified by decision tree *M *as positive

### Feature construction by clustering malignant cells (positive pixels)

Cluster analysis is an important data mining task for finding meaningful groups in a dataset. In the literature, various clustering algorithms including density-based, graph-based, hierarchical, and K-means are applied to diverse applications such as web mining, pattern recognition, fraud detection, and meteorology [20].

From the standpoint of the malignant cell clustering, we redefine and make use of a density-based approach, namely DBSCAN (A Density-Based Algorithm for Discovering Clusters in Large Spatial Databases with Noise) [14]. More specifically, DBSCAN is a density-based clustering method, which was designed to detect clusters of arbitrary shape as well as to distinguish noise in spatial and multi-dimensional databases. DBSCAN is significantly more effective in discovering clusters of arbitrary shape which perfectly fits the task of detection of malignant areas. It was successfully used for synthetic dataset as well as earth science, and protein dataset [14]. We omit theoretical background of DBSCAN and refer the reader to [14] for detailed explanations.

In this subsection, we introduce the notion of malignant cells that are composed of and connected via positive colors. Technically, it is appropriate to adapt DBSCAN in which cluster definition guarantees that the number of positive pixels is equal to or greater than a threshold in certain neighborhood of selected pixels. However, it is necessary to revisit density-based clustering algorithm so that we can tailor this approach to our framework. Let *I *be a subimage that is of dimension 128 × 128 and comprises of pixels whose labels are either positive or negative (see Definition 4). For a particular pixel *p*, let *p*_*x *_and *p*_*y *_denote its position at *x*^*th *^column and *y*^*th *^row with respect to top-left corner *(0, 0) *of subimage *I*. Let *p*_*xy *_represent the color at (*p*_*x*_, *p*_*y*_).

Definition 5 (*ε*-neighborhood of a pixel *p*, *M*(*p*) = positive): The *ε*-neighborhood of a pixel p, denoted by *ε*(*p*), is defined as *ε *(*p*) = {*q *∈ *I *| (*d*(*p*, *q*) ≤ *ε*) ∧ (*M*(*q*) = *positive*)} where *d*(*p*, *q*) is Euclidean distance between two pixels and denoted by d(p,q)=(px−qx)2+(py−qy)2
 MathType@MTEF@5@5@+=feaafiart1ev1aaatCvAUfKttLearuWrP9MDH5MBPbIqV92AaeXatLxBI9gBaebbnrfifHhDYfgasaacH8akY=wiFfYdH8Gipec8Eeeu0xXdbba9frFj0=OqFfea0dXdd9vqai=hGuQ8kuc9pgc9s8qqaq=dirpe0xb9q8qiLsFr0=vr0=vr0dc8meaabaqaciaacaGaaeqabaqabeGadaaakeaacqWGKbazcqGGOaakcqWGWbaCcqGGSaalcqWGXbqCcqGGPaqkcqGH9aqpdaGcaaqaaiabcIcaOiabdchaWnaaBaaaleaacqWG4baEaeqaaOGaeyOeI0IaemyCae3aaSbaaSqaaiabdIha4bqabaGccqGGPaqkdaahaaWcbeqaaiabikdaYaaakiabgUcaRiabcIcaOiabdchaWnaaBaaaleaacqWG5bqEaeqaaOGaeyOeI0IaemyCae3aaSbaaSqaaiabdMha5bqabaGccqGGPaqkdaahaaWcbeqaaiabikdaYaaaaeqaaaaa@4949@ and *ε *is distance threshold between two pixels. *M *is the induced decision tree for the classification of pixels. As for respective pixels in a potential cluster, a well-designed approach would require that there is at least a minimum number (MinPxl) of qualified pixels in *ε*(*p*), since malignancy is seen in the form of well-connected and dense positive pixels. Hence, we look for another condition in *ε*(*p*): | *ε *(*p*) | ≥ *MinPxl*. This condition ensures that peripheral positive pixels are quantitatively sufficient to form a malignant cell structure. As is clear from above definitions, in order to find malignant regions, the algorithm starts with a randomly selected pixel *p*, whose color is of malignant, *M*(*p*) = positive. After that, as the second condition | *ε *(*p*) | ≥ *MinPxl *is satisfied, a new cluster is highlighted in the neighborhood of pixel *p*, which is now center of new nuclei region. In the following we basically present the algorithm which starts with a subimage and parameters of *ε *and *MinPxl*:

ALGORITM:DBSCAN

DBSCAN(SubImage, Eps, MinPxl)

ClusterId : = nextId(NOISE);

FOR i FROM 1 TO SubImage.height DO

   FOR j FROM 1 TO SubImage.width DO

      Pxl : = SubImage.get(i, j);

      IF Pxl.C_Id = UNCLASSIFIED AND M(Pxl) = positive

      THEN

         IF DilateCluster(SubImage, Pxl, ClusterId, Eps, MinPxl)

         THEN

            ClusterId : = nextId(ClusterId)

         END IF;

      END IF;

   END FOR;

   END FOR;

END DBSCAN;

Throughout the main procedure of DBSCAN we try to expand current cluster by inspecting the other positive pixels within newly formed cluster. If another cluster whose center pixel *q *is inside of *ε*(*p*) is detected, these two clusters are merged instead of constructing a new one. These operations are accomplished by the function DilateCluster, which is most important subfunction used by DBSCAN and given below:

SUBPROCEDURE:DILATECLUSTER

DilateCluster(SubImage, Pxl, C_Id, Eps, MinPxl): Boolean;

   cand_cls: = regionQuery(Pxl, Eps);

   IF cand_cls.size < MinPxl THEN // no core point

      set_C_Id(Pxl, NOISE);

      RETURN False;

      ELSE // all pixels in seeds are density reachable from current point

      change_C_Ids(cand_cls, C_Id);

      cand_cls.remove(Pxl);

      WHILE cand_cls <> Empty DO

         Cur_Pxl: = cand_cls.first();

         exp_set: = regionQuery(Cur_Pxl, Eps);

         IF exp_set.size >= MinPxl

            FOR i FROM 1 TO exp_set.size() DO

               NPxl: = exp_set.get(i);

               IF NPxl.C_Id IN {UNCLASSIFIED, NOISE} THEN

                  IF NPxl.C_Id = UNCLASSIFIED THEN

                     IF M(NPxl) = positve THEN

                        cand_cls.add(NPxl);

                     END IF;

                  END IF;

               set_C_Id(NPxl, C_Id);

               END IF; // UNCLASSIFIED or NOISE

            END FOR;

         END IF; // exp_set.size >= MinPxl

         cand_cls.delete(Cur_Pxl);

      END WHILE; // seeds <> Empty

      RETURN True;

   END IF;

END; // DilateCluster

**Figure 7:**
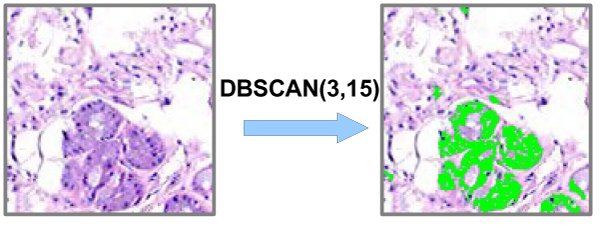
**Original image (left), and image (right) with colored cell nuclei by DBSCAN (*ε = 3, MinPxl = 15*)**. The density-based clustering algorithm (DBSCAN) is used to detect positive color areas, which is green area in the figure. The positive color areas are cell nuclei, which build the features for classification.

A call of regionQuery(Pxl, Eps) returns *ε*-neighborhood of the pixel in current subimage as a list of pixels. The C_Id of pixels which have been marked to be NOISE may be changed later, if they are contained in other clusters of subimage. Basically speaking unclustered pixels are of either negative colors or positive colors that cannot form a cluster of malignant cell(s) spatially because of their sparsity. The phase of clustering is ended once all positive color pixels are evaluated by the algorithm. It is worth to note that a subimage that has clustered cell regions, as in Figure 7, cannot be considered directly being malignant. The algorithm detailed in this section is used to detect cell structures that are stained with haematoxylin. The reason is that haematoxylin colors all cell nuclei including both malignant and non-malignant.

Recall that despite the fact that existence of the clusters resulted from DBSCAN in a subimage can affect likelihood of having malignant cells, the characteristics of discovered regions should be further investigated carefully in order to make final decision about a subimage whether it belongs to malignant or healthy class. To be used within classification schema, we only discretize the histogram of positive colors that are members of the clusters. Please note that the pixels inside the clusters are of positive colors, but not vice versa. Considering hue, saturation, and value components of a color *c*, histogram discretization is performed based on only hue and saturation because value shows wide range of color distribution in both malignant and healthy cells. The histograms of hue and saturation values are partitioned to obtain 24 equi-width bins. This process results in a feature vector that is represented by a set of bins, where each bin presents frequency of colors that fall into it.

### Delineation of malignancy

The previous sections describe the method to map each training subimages into a 24-dimensional feature vectors. In this section, we describe the training of a classifier to classify the subimages represented by feature vectors. Support Vector Machines (SVMs) [17] have been successfully and widely applied to various real-world classification problems, such as face detection [21], three-dimensional (3-D) objects recognition [22], and so on [17]. Thus, in order to classify subimages, SVMs were preferred for its robustness and good performance to tackle our two-class classification problem. For the sake of brevity, we omit the theoretical background of SVMs, and refer the reader to [17].

In the learning phase, two-class SVMs classifier is trained with radial base kernel and based on total of 955 training subimages. The search of good parameters for the SVMs classifier is done by evaluating candidate sets from the parameter space. For each set of parameters, we conduct ten-fold cross-validation on training subimage. The configuration with the best cross-validation performance S* is reserved. The training SVMs is then applied to classify all the subimages to delineate malignant areas.

Depending on accuracy of the results, the weights of the classes can be adjusted to build more conservative or tolerant classifier. Consequently, this introduces an appealing trade-off between recall and precision for the slides with cancer regions.

## Competing interests

The University of Arkansas for Medical Sciences has applied for a US patent that utilized the technology described in the manuscript. Drs. Shafirstein, Xu and Mr. Mete are the co-inventors in this patent application. Dr. Shafirstein also has interest in the commercial application of this technology.

## Authors' contributions

GS has conceived the study. All of the authors participated in the overall design of the study. CYF described the visual details of tumor and marked the malignant regions in the slides. MM and XX designed the algorithms, MM developed the software and performed data analysis, algorithm testing, and benchmarking. XX and MM wrote the manuscript.
